# Spatial separation of phosphatase and kinase activity within the Bub complex is required for proper mitosis

**DOI:** 10.1093/jmcb/mjac062

**Published:** 2022-11-28

**Authors:** Lei Wang, Thomas Kruse, Blanca López-Méndez, Yuqing Zhang, Chunlin Song, Lei Zhu, Bing Li, Jing Fang, Zhimin Lu, Jakob Nilsson, Gang Zhang

**Affiliations:** The Cancer Institute, The Affiliated Hospital of Qingdao University, Qingdao University, Qingdao 266061, China; Novo Nordisk Foundation Center for Protein Research, Faculty of Health and Medical Sciences, University of Copenhagen, Copenhagen DK-2200, Denmark; Novo Nordisk Foundation Center for Protein Research, Faculty of Health and Medical Sciences, University of Copenhagen, Copenhagen DK-2200, Denmark; The Cancer Institute, The Affiliated Hospital of Qingdao University, Qingdao University, Qingdao 266061, China; The Cancer Institute, The Affiliated Hospital of Qingdao University, Qingdao University, Qingdao 266061, China; The Cancer Institute, The Affiliated Hospital of Qingdao University, Qingdao University, Qingdao 266061, China; The Department of Genetics and Cell Biology, Basic Medical College, Qingdao University, Qingdao 266061, China; The Cancer Institute, The Affiliated Hospital of Qingdao University, Qingdao University, Qingdao 266061, China; Institute of Translational Medicine, Zhejiang University School of Medicine, Hangzhou 310029, China; Novo Nordisk Foundation Center for Protein Research, Faculty of Health and Medical Sciences, University of Copenhagen, Copenhagen DK-2200, Denmark; The Cancer Institute, The Affiliated Hospital of Qingdao University, Qingdao University, Qingdao 266061, China; Novo Nordisk Foundation Center for Protein Research, Faculty of Health and Medical Sciences, University of Copenhagen, Copenhagen DK-2200, Denmark

**Keywords:** mitosis, spindle assembly checkpoint, kinetochores, Bub1, BubR1, PP2A/B56

## Abstract

The Bub1 and BubR1 kinetochore proteins support proper chromosome segregation and mitotic checkpoint activity. Bub1 and BubR1 are paralogs with Bub1 being a kinase, while BubR1 localizes the PP2A-B56 protein phosphatase to kinetochores in humans. Whether this spatial separation of kinase and phosphatase activity is important is unclear as some organisms integrate both activities into one Bub protein. Here, we engineer human Bub1 and BubR1 proteins integrating kinase and phosphatase activities into one protein and show that these do not support normal mitotic progression. A Bub1–PP2A-B56 complex can support chromosome alignment but results in impairment of the checkpoint due to dephosphorylation of the Mad1 binding site in Bub1. Furthermore, a chimeric BubR1 protein containing the Bub1 kinase domain induces delocalized H2ApT120 phosphorylation, resulting in the reduction of centromeric hSgo2 and chromosome segregation errors. Collectively, these results argue that the spatial separation of kinase and phosphatase activities within the Bub complex is required for balancing its functions in the checkpoint and chromosome alignment.

## Introduction

The accurate segregation of the genetic material during cell division requires that kinetochores establish proper connections to the microtubules of the mitotic spindle. A complex surveillance mechanism, the spindle assembly checkpoint (SAC), monitors kinetochore–microtubule interactions and delays mitotic exit until all kinetochores have bound to microtubules (Lara-Gonzalez et al., 2012, 2021b; Musacchio, 2015). The SAC is composed of a set of conserved checkpoint proteins that associates with outer kinetochore proteins during prometaphase to generate a ‘wait anaphase’ signal. Generation of the SAC signal depends on the assembly of a Mad1/2–Bub1 complex, which is mediated by Mps1 phosphorylation of Bub1 Thr 461 (T461) in humans (Brady and Hardwick, 2000; London and Biggins, 2014; Moyle et al., 2014; Faesen et al., 2017; Ji et al., 2017; Zhang et al., 2017). In turn, the Mad1/2–Bub1 complex stimulates the generation of the mitotic checkpoint complex (MCC) composed of the BubR1 and Mad2 checkpoint proteins bound to Cdc20 (Sudakin et al., 2001; Faesen et al., 2017; Ji et al., 2017; Piano et al., 2021; Lara-Gonzalez et al., 2021a). The MCC constitutes the biochemical identity of the ‘wait anaphase’ signal.

**Figure 1:**
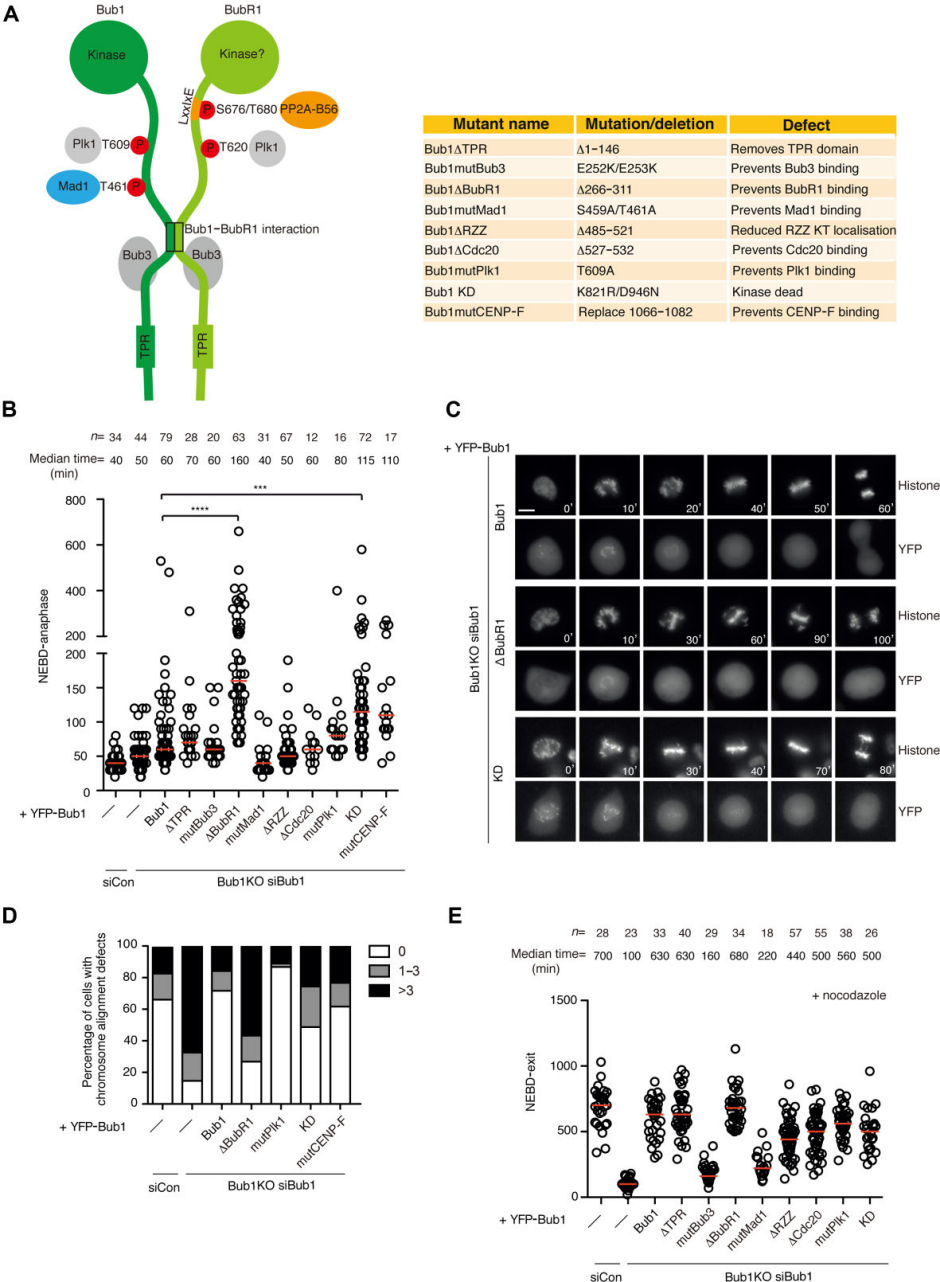
Systematic analysis of Bub1 interaction modules. (**A**) Schematic of the Bub1–BubR1 complex and a table of the Bub1 mutants analyzed in this study. TPR, tetratricopeptide repeat. (**B**) The time from NEBD to anaphase in HeLa cells (siCon) or Bub1KO cells complemented with the indicated YFP-tagged Bub1 constructs after depletion of endogenous Bub1. Each circle represents the timing of a single cell and the red line indicates the median time. Mann–Whitney U-test was applied. ****P < 0.0001, ***P < 0.001. The number of cells analyzed for each condition is indicated on top. Representative experiment of two independent experiments of each mutant except for Bub1ΔBubR1 and Bub1 KD, which were repeated at least three times. (**C**) Representative still images of mitosis in Bub1KO cells complemented with YFP-tagged Bub1, Bub1ΔBubR1, Bub1 KD, and CFP-H3 constructs after depletion of endogenous Bub1. The top panel is CFP-H3 and the bottom panel is YFP-Bub1. Scale bar, 5 μm. (**D**) Quantification of the chromosome alignment defects in MG132-arrested HeLa cells (siCon) or Bub1KO cells complemented with the indicated YFP-tagged Bub1 constructs after depletion of endogenous Bub1. Cells were stained with DAPI and antibodies for tubulin, YFP, and CENP-C, and the number of unaligned chromosomes was counted. A hundred cells were analyzed per condition and the experiment was performed twice. Results from one of the experiments are shown. (**E**) The time from NEBD to mitotic exit of nocodazole-treated HeLa cells (siCon) or Bub1KO cells complemented with the indicated YFP-tagged Bub1 constructs after depletion of endogenous Bub1. Each circle represents the time from NEBD to mitotic exit of a single cell and the red line indicates the median time. The number of cells analyzed per condition is indicated above. Representative experiment of two independent experiments is shown.

In addition to generating a checkpoint signal in response to unattached kinetochores, several checkpoint proteins facilitate the establishment of proper kinetochore–microtubule interactions. In particular, the Bub1 and BubR1 checkpoint proteins have been shown to be important for alignment of chromosomes independent of their checkpoint function (Lampson and Kapoor, 2005; Meraldi and Sorger, 2005; Elowe et al., 2010; Elowe and Bolanos-Garcia, 2022). Bub1 and BubR1 are paralogs that arose from gene duplications of an ancestral ‘MadBub’ gene (Vleugel et al., 2012; Tromer et al., 2016; Kops et al., 2020). Bub1 and BubR1 share a common GLEBS interaction motif for Bub3 and directly bind to each other through a region C-terminal to the Bub3 binding region (Figure 1A; Taylor et al., 1998; Overlack et al., 2015; Zhang et al., 2015). In addition, numerous short linear motifs (SLiMs) are present throughout the Bub1 and BubR1 proteins that mediate interactions with specific mitotic regulators (Figure 1A; Elowe and Bolanos-Garcia, 2022). At the C-terminus, both proteins contain a kinase domain that in the case of BubR1 is argued to be either a pseudokinase (Suijkerbuijk et al., 2012a) or an active kinase (Huang et al., 2019; Tang et al., 2020), while Bub1 is an active kinase that phosphorylates T120 on histone H2A to facilitate hSgo1 and hSgo2 recruitment to centromeres (Kawashima et al., 2010; Yamagishi et al., 2010).

BubR1 supports chromosome alignment by recruiting the PP2A-B56 Ser/Thr protein phosphatase through a well-characterized LxxIxE motif to counterbalance Aurora B and Plk1 kinase activity at kinetochores (Lampson and Kapoor, 2005; Foley et al., 2011; Suijkerbuijk et al., 2012b; Kruse et al., 2013; Zhang et al., 2015; Hertz et al., 2016). In addition, BubR1 might phosphorylate CENP-E to regulate lateral to end-on capture of microtubules (Huang et al., 2019). Several functions of Bub1 in chromosome alignment have also been uncovered, including kinetochore recruitment of Plk1 and CENP-F, binding to BubR1 as well as Bub1 kinase activity (Elowe et al., 2007; Berto et al., 2018; Nguyen et al., 2021; Singh et al., 2021; Elowe and Bolanos-Garcia, 2022). Recent data have suggested that Plk1 recruitment by Bub1 is redundant due to CENP-U recruitment of Plk1 (Chen et al., 2021; Nguyen et al., 2021; Singh et al., 2021). However, the relative contribution of the Bub1 interactions to chromosome alignment and SAC function is unclear. This is in part because the investigation of Bub1 function is challenging in human cells, as very low level of Bub1 is sufficient for its function (Rodriguez-Rodriguez et al., 2018; Meraldi, 2019; Zhang et al., 2019). We recently developed an approach that circumvents this problem by combining CRISPR/Cas9-mediated knockout (KO) of Bub1 and RNAi depletion of residual Bub1, allowing functional investigations into Bub1 (Zhang et al., 2019). We here use this approach to perform a systematic analysis and side-by-side comparison of the different functional domains and SLiMs in Bub1 and their contribution to chromosome alignment and SAC function. This provided novel insights into Bub1 function and revealed a requirement for separation of kinase and phosphatase activities in the Bub1–BubR1 complex for accurate chromosome segregation in human cells.

## Results

### A systematic analysis of Bub1 functional modules

We recently used CRISPR/Cas9 to target Bub1 in HeLa cells, hereby generating a cell line with low levels of endogenous Bub1 (Zhang et al., 2019). Depletion of the residual Bub1 by RNAi results in almost complete removal of Bub1 with penetrant phenotypes in chromosome alignment and SAC signaling. By complementing Bub1-depleted cells with an RNAi-resistant YFP-tagged Bub1 construct, these phenotypes are suppressed providing an opportunity for analyzing the effects of Bub1 mutations in a clean null background. Numerous functional protein–protein interaction modules in Bub1 have been reported to regulate SAC signaling and chromosome alignment (Figure 1A). However, these modules have not all been analyzed in a clean Bub1 null background, making unambiguous comparisons difficult. Therefore, we decided to do a systematic side-by-side analysis of the modules of Bub1 to investigate their respective contribution to Bub1 function.

We combined cell synchronization with Bub1 RNAi depletion and complementation with YFP-tagged Bub1 constructs and followed mitotic progression by time-lapse microscopy. We recorded time from nuclear envelope breakdown (NEBD) to anaphase in single cells and monitored chromosome alignment using a fluorescent histone variant (Figure 1B and C). We only analyzed cells that expressed similar levels of exogenous Bub1 proteins as judged by the YFP signal. All Bub1 mutants analyzed localized efficiently to kinetochores both by live cell microscopy and by immunofluorescence except for the Bub1mutBub3 (Supplementary Figure S1A). In the complete absence of Bub1, both chromosome congression and the SAC are severely impaired, resulting in cells exiting mitosis after ∼50 min with multiple unaligned chromosomes. The alignment defect was fully rescued by expressing YFP-Bub1 showing that our complementation assay reports on Bub1 functionality. From our analysis on Bub1 mutants, the disruption of three functional modules in Bub1 increased mitotic timing and induced chromosome alignment defects: the region binding to BubR1, the Bub1 kinase activity, and the C-terminal helix reported to bind to CENP-F (Figure 1B and C). The important role of these functional modules was confirmed by immunofluorescence analysis of chromosome alignment in cells treated with a proteasome inhibitor (Figure 1D). However, subsequent in-depth analysis of our Bub1 CENP-F mutant, which replaced the C-terminal helix in Bub1 with an irrelevant helix, revealed that it was defective in kinase activity, and we therefore could not access the direct contribution of the Bub1–CENP-F interaction to chromosome congression (Supplementary Figure S1B and C). Given that this helix is an integral component of the Bub1 kinase domain, it is not surprising that kinase activity is lost in this mutant (Kang et al., 2008). We note that the GLEBS domain is essential for Bub1 function, but due to the lack of a functional checkpoint in this mutant, mitotic timing is not increased.

To analyze the effect on SAC signaling, we used a similar experimental set-up and challenged cells with nocodazole to activate the checkpoint and recorded time from NEBD to mitotic exit (Figure 1E). This revealed a strong requirement for the Bub3 interaction domain as well as the Mad1 interaction domain as expected. We observed only a minor contribution to SAC signaling from the ABBA motif that binds Cdc20 (Di Fiore et al., 2015), suggesting that this interaction is not essential for the SAC in human cells in contrast to *Caenorhabditis elegans* and *in vitro* biochemical reconstituted assays (Piano et al., 2021; Lara-Gonzalez et al., 2021a).

In summary, we have provided a careful analysis of the functional determinants of Bub1, revealing their relative contribution to chromosome alignment and SAC signaling. Bub1 interactions to Bub3 and Mad1 seemed to be the most important for SAC signaling. For chromosome alignment, the binding between Bub1 and BubR1 played the major role, and the Bub1 kinase activity was also required.

### Recruitment of PP2A-B56 to Bub1 supports chromosome alignment

**Figure 2:**
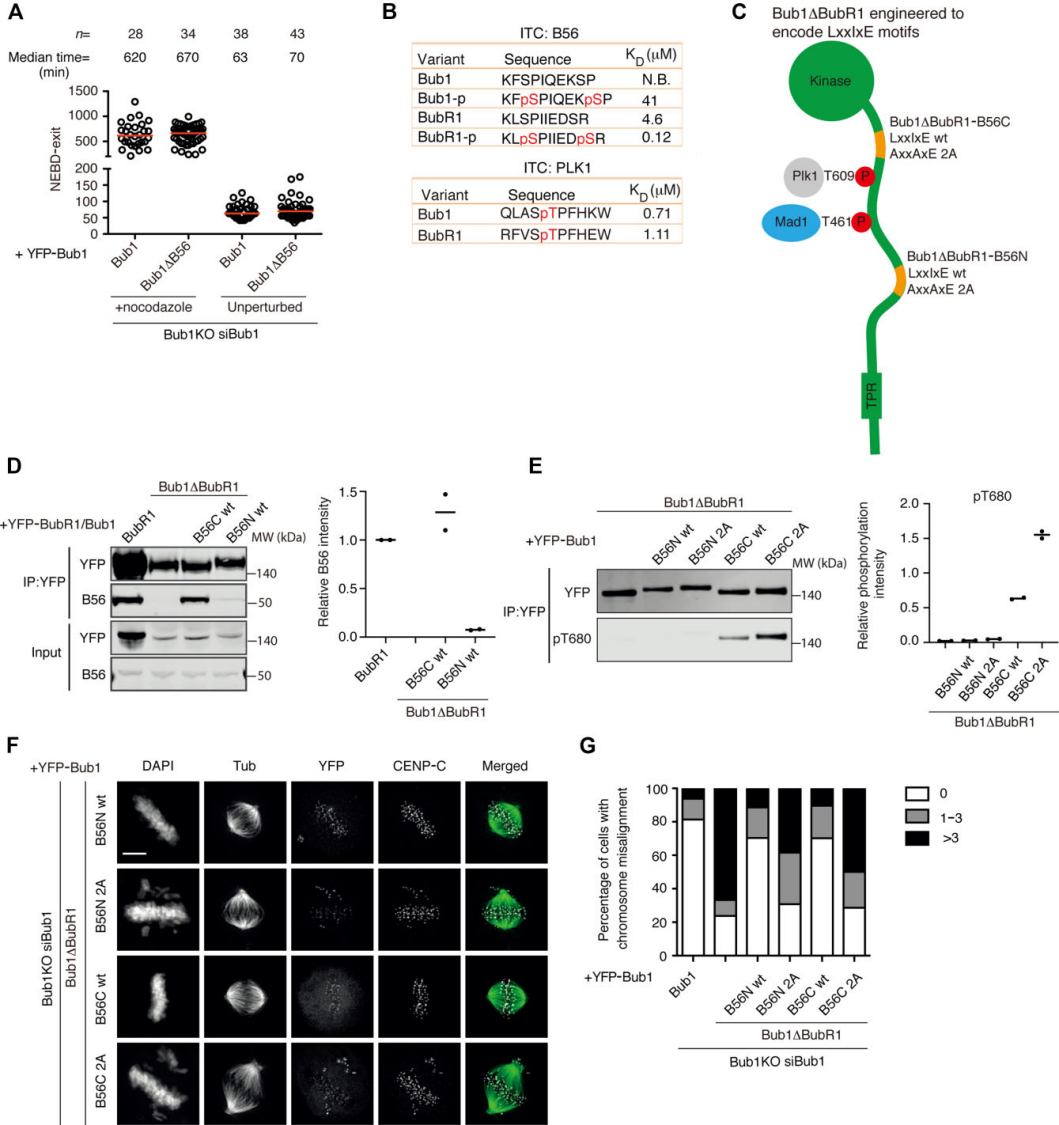
Engineered Bub1 proteins bind to PP2A-B56. (**A**) The time from NEBD to mitotic exit of nocodazole-treated Bub1KO cells or from NEBD to anaphase of untreated Bub1KO cells complemented with the indicated YFP-tagged Bub1 constructs after depletion of endogenous Bub1. Each circle represents the time a single cell spending in mitosis and the red line indicates the median time. The number of cells analyzed per condition is indicated above. Representative experiment of two independent experiments is shown. (**B**) Affinity measurements of Bub1 and BubR1 peptides binding to B56α or the Plk1 polobox domain. The affinities for BubR1 peptides binding to B56α are from Kruse et al. (2013). (**C**) Schematic of the position of the LxxIxE motifs in the engineered Bub1 proteins. (**D**) Quantitative western blotting analysis of YFP immunoprecipitates of the indicated YFP-tagged proteins probed for B56α and YFP. The level of B56α was normalized to that of YFP in the precipitated samples. Quantification of two repeats is presented on the right with the level in YFP-BubR1 set to 1. (**E**) Quantitative western blotting analysis of the phosphorylation on BubR1 T680 in the immunoprecipitates of the indicated YFP-tagged proteins. The level of T680 phosphorylation was normalized to that of YFP in the precipitated samples. Quantification of two repeats is presented on the right. (**F** and **G**) Representative images of MG132-arrested Bub1KO cells expressing Bub1ΔBubR1-B56N and Bub1ΔBubR1-B56C constructs after depletion of endogenous Bub1. Fixed cells were stained by DAPI and antibodies for tubulin, YFP, and CENP-C. The cells were analyzed by microscopy, and chromosome alignment defects were examined. A hundred cells were analyzed per condition. Representative experiment of two independent experiments is shown. Scale bar, 5 μm.

Based on the above results, we decided to investigate the role of the BubR1–Bub1 interaction in more detail. Since it is well established that BubR1 recruits PP2A-B56 to facilitate proper kinetochore–microtubule interactions, we speculated that the lack of kinetochore localized PP2A-B56 in Bub1ΔBubR1 complemented cells was the cause of alignment defects. In cells with Bub1 completely removed, there was ∼30% of BubR1 remained on kinetochores compared to parental cells. Interestingly, the level of BubR1 pT680, constituting part of the PP2A-B56 binding site, was reduced to 5%, indicating that a stable interaction of BubR1–Bub1 is required for the efficient phosphorylation on this site. In Bub1ΔBubR1 complemented cells, ∼20% endogenous BubR1 remained on kinetochores (Supplementary Figure S2A–C; Supplementary Figure S3A). This argues that insufficient levels of BubR1 and PP2A-B56 are recruited to kinetochores in Bub1ΔBubR1-complemented cells. Consistent with this idea, we observed increased kinetochore phosphorylation in Bub1ΔBubR1-complemented cells (Supplementary Figure S2D). However, human Bub1 also contains a putative PP2A-B56 binding motif (amino acids 654-FSPIQE-659) and Bub1 in *C. elegans* has been shown to bind directly to PP2A-B56 (Bel Borja et al., 2020). We therefore first investigated whether the PP2A-B56 binding motif in human Bub1 was functionally important. Using our Bub1 complementation approach, we observed no effects on chromosome alignment or SAC activity when we deleted 634–686 aa, which encompasses the putative Bub1 PP2A-B56 binding motif (Bub1ΔB56) (Figure 2A). Consistently, isothermal titration calorimetry (ITC) measurements of Bub1 peptides encompassing the binding motif showed very weak binding to B56α (Figure 2B; Supplementary Figure S2E and F). Indeed, the affinity was almost decreased 400-fold compared to the BubR1 PP2A-B56 binding motif (Kruse et al., 2013). Furthermore, immunoprecipitation experiments showed that Bub1ΔBubR1 from mitotic cells did not interact with PP2A-B56 (Figure 2D; Supplementary Figure S3B). We thus conclude that Bub1 does not encode a functionally PP2A-B56 binding motif in human cells.

If the main function of the Bub1–BubR1 interaction in chromosome alignment is to recruit PP2A-B56 to kinetochores, one prediction would be that engineering a PP2A-B56 binding motif into Bub1ΔBubR1 would rescue chromosome alignment defects. To test this, we engrafted residues 649–697 from BubR1, which encompasses its PP2A-B56 LxxIxE binding motif into two distinct positions of Bub1ΔBubR1 (Figure 2C). In one engineered Bub1 protein, we engrafted the region into the BubR1 binding site of Bub1 (Bub1ΔBubR1-B56N). In the other, we replaced the non-functional B56 motif of Bub1 with that of BubR1 (Bub1ΔBubR1-B56C). In these mutants, the same low level of endogenous BubR1 is recruited to kinetochores (Supplementary Figure S3A). As a control, we generated the same engineered Bub1 proteins but with two amino acid mutations in the PP2A-B56 binding site (Bub1ΔBubR1-B56N/C 2A, LxxIxE mutated to AxxAxE). We first tested whether these engineered Bub1 proteins bind PP2A-B56 by immunopurifying YFP-tagged Bub1 proteins from mitotic cells. We observed a strong interaction between Bub1ΔBubR1-B56C and PP2A-B56 and the amount co-purified was comparable to that co-purified with BubR1 (Figure 2D). The Bub1ΔBubR1-B56N also bound to PP2A-B56 but to a much lesser extent. The interaction to PP2A-B56 was abolished in both Bub1ΔBubR1-B56 2A constructs (Supplementary Figure S3B). A molecular explanation for why Bub1-B56C binds to more PP2A-B56 is possibly the close proximity of the engrafted BubR1 fragment to the Plk1 docking site (T609) in Bub1 (Qi et al., 2006; Singh et al., 2021). The interaction between PP2A-B56 and BubR1 is strongly stimulated by Plk1 phosphorylation of BubR1 S676 and T680 with Plk1 binding to T620 in BubR1 (Elowe et al., 2007; Suijkerbuijk et al., 2012b; Kruse et al., 2013). The binding of Plk1 to T609 in Bub1 is very similar to T620 in BubR1, and since the two Plk1 binding motifs bind with similar affinity to the Plk1 polo box domain (Figure 2B; Supplementary Figure S3C and D), this would likely result in efficient S676 and T680 phosphorylation in Bub1ΔBubR1-B56C. Consistent with this, we observed a high level of T680 phosphorylation specifically in Bub1ΔBubR1-B56C, which was abolished by the mutation of T609 (Figure 2E; Supplementary Figure S3E and F).

**Figure 3:**
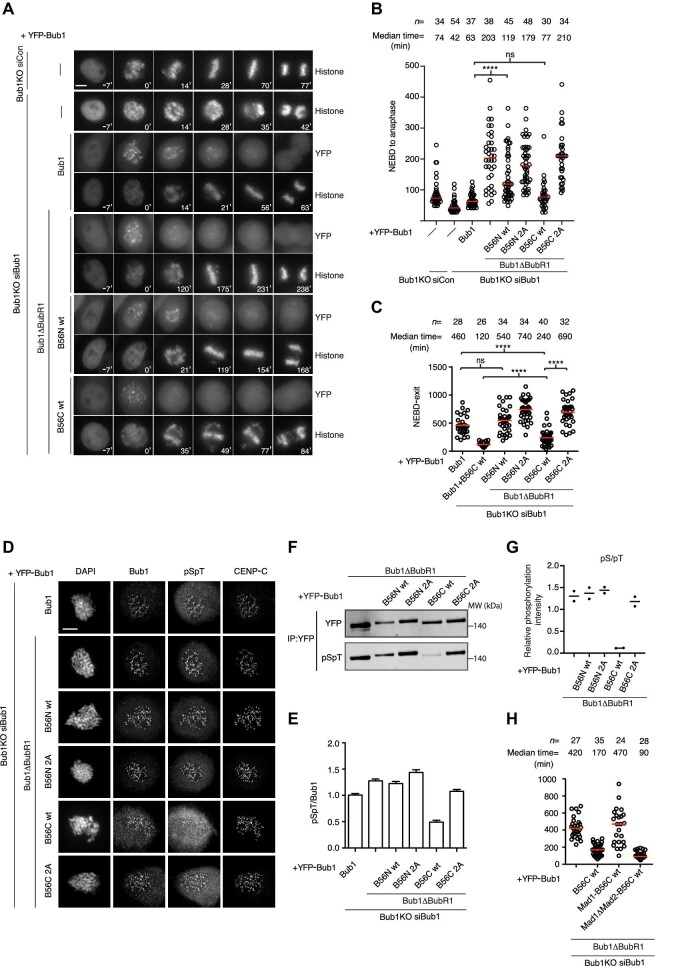
Recruitment of PP2A-B56 to Bub1 disrupts SAC activity. (**A**) Representative still images of mitosis of Bub1KO cells complemented with the indicated YFP-tagged Bub1 constructs and a CFP-tagged histone marker after depletion of luciferase as control or endogenous Bub1. (**B**) The time from NEBD to anaphase in Bub1KO cells as in **A**. Pooled data from three independent experiments. (**C**) The time from NEBD to exit in nocodazole-treated Bub1KO cells supplemented with YFP-tagged Bub1 constructs after depletion of endogenous Bub1. Representative experiment of three independent experiments is shown. For **B** and **C**, each circle represents the time spending in mitosis of a single cell. Red line indicates the median time, and the number of cells analyzed per condition is indicated above. Mann–Whitney U-test was applied. ns, not significant. ****P < 0.0001. (**D**) Representative images of nocodazole-arrested Bub1KO cells complemented with the indicated YFP-Bub1 constructs after depletion of endogenous Bub1 and stained with DAPI and antibodies for Bub1, Bub1 pS459/pT461 (pSpT), and CENP-C. (**E**) Quantification of the kinetochore signal of Bub1 pS459/pT461 normalized to that of Bub1 in **D**. At least 150 kinetochores from 10 cells were quantified and plotted for each condition. Bar indicates mean, and standard error of the mean (SEM) is shown by a line. The experiment was repeated twice, and the result of one experiment is shown. (**F** and **G**) Quantitative western blotting analysis of Bub1 pS459/pS461 in the indicated YFP-Bub1 proteins purified from nocodazole-arrested HeLa cells. The level of pSpT/YFP from two repeats is shown in **G**. (**H**) The time from NEBD to mitotic exit in nocodazole-treated Bub1KO cells complemented with the indicated YFP-tagged Bub1 constructs after depletion of endogenous Bub1. Each circle represents the time spent in mitosis of a single cell. Red line indicates the median time, and the number of cells analyzed per condition is indicated above. Representative experiment of two independent experiments is shown. Scale bar, 5 μm.

To test whether the engineered Bub1 proteins supported chromosome alignment, we first analyzed this using immunofluorescence assays. In these assays, we added a proteasome inhibitor, hereby avoiding any indirect effects from changes to SAC signaling in the different Bub1 proteins. We scored unaligned chromosomes in individual cells, which revealed that engrafting a functional PP2A-B56 binding site into Bub1ΔBubR1 supported chromosome alignment (Figure 2F and G). Next, we analyzed these mutants by live cell microscopy and recorded both mitotic timing and defects in chromosome alignment. In this experimental set-up, we are monitoring the contribution of Bub1 to SAC activity and chromosome alignment. In both Bub1ΔBubR1-B56N/C, we observed that mitotic timing was reduced compared to Bub1ΔBubR1, and this reduction was dependent on a functional PP2A-B56 binding site (Figure 3A and B). However, Bub1ΔBubR1-B56N-complemented cells still spent longer time in mitosis compared to Bub1 wild-type (wt)-complemented cells. Further analysis revealed a delay in chromosome alignment in these cells, whereas they only entered anaphase with fully aligned chromosomes indicating a functional SAC. In contrast, Bub1ΔBubR1-B56C-complemented cells exited mitosis with a similar timing to Bub1 wt-complemented cells but with unaligned chromosomes, suggesting failure in proper SAC signaling.

Collectively, these data support the conclusion that recruitment of PP2A-B56 to Bub1 can support the alignment of chromosomes but not as efficient as when PP2A-B56 is recruited to kinetochores via BubR1.

### Recruitment of PP2A-B56 to Bub1 results in SAC failure due to dephosphorylation of the Mad1 binding site

The fact that Bub1ΔBubR1-B56C-complemented cells exited mitosis with unaligned chromosomes argued for an underlying defect in SAC signaling. Indeed, challenging cells with nocodazole confirmed a specific SAC defect in Bub1ΔBubR1-B56C (Figure 3C). This defect in SAC signaling was even more pronounced in cells complemented with a Bub1 construct (Bub1 + B56C wt) that maintained its interaction with BubR1 and thus recruits PP2A-B56 both through BubR1 and through the engineered LxxIxE motif in the Bub1 C position (Figure 3C). To investigate the molecular basis for this, we focused on the Bub1 phosphorylation sites critical for SAC function since we anticipated that they might be dephosphorylated by the PP2A-B56 bound to Bub1. Bub1 phosphorylation by Mps1 on T461 creates a binding site for Mad1 on Bub1 required for SAC signaling. We accessed the phosphorylation status of this site using a phospho-specific antibody (pSpT) (Zhang et al., 2017). By performing immunopurification and immunofluorescence staining, we observed a clear reduction in phosphorylation of this site in Bub1ΔBubR1-B56C, which was dependent on PP2A-B56 binding (Figure 3D–G). This would argue that the underlying molecular defect in SAC signaling in Bub1ΔBubR1-B56C is a disruption of the Bub1–Mad1 interaction. Indeed, if we fused Mad1 485–715 aa to the N-terminus of Bub1ΔBubR1-B56C (Mad1-B56C wt), we could suppress the SAC defect (Figure 3H). As a control, the fusion of Mad1 485–715 aa mutant defective in binding to Mad2 did not suppress the SAC defect.

These results show that efficient binding of PP2A-B56 to Bub1 is not compatible with a functional SAC, resulting in chromosome segregation defects. However, low levels of PP2A-B56 recruitment to Bub1 as in Bub1ΔBubR1-B56N do not affect SAC signaling, but these levels are insufficient for normal timing of mitotic progression.

### A chimeric BubR1 protein with Bub1 kinase activity does not support proper chromosome segregation

**Figure 4:**
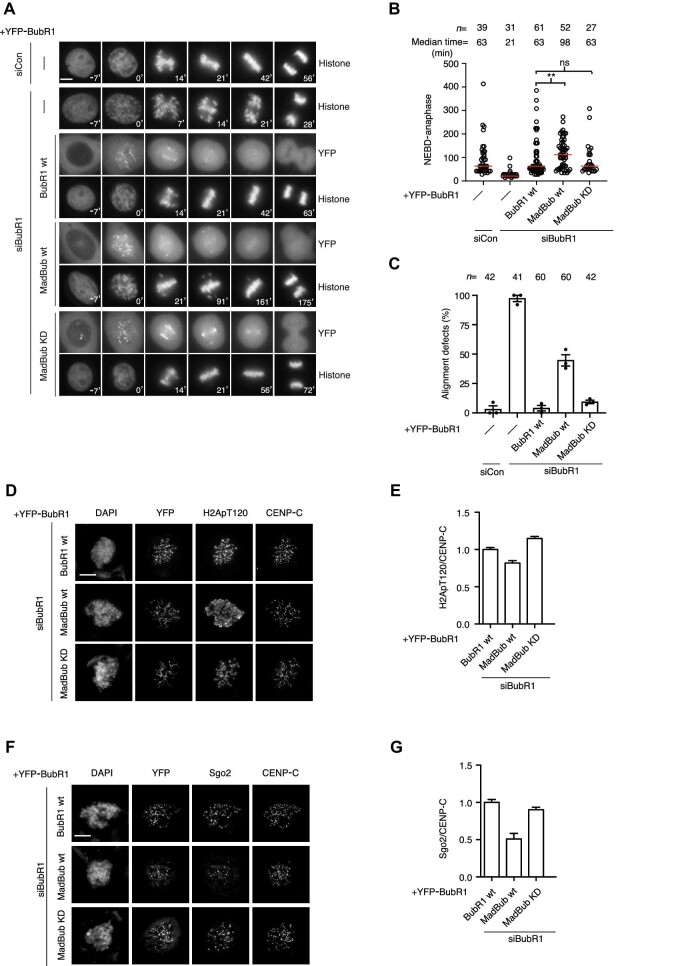
Restoring BubR1 kinase activity disrupts accurate mitosis. (**A**) Representative time-lapse images of unperturbed mitosis in HeLa cells complemented with the indicated YFP-tagged BubR1 proteins (wt or MadBub) and CFP-H3 after depletion of luciferase as control or endogenous BubR1. (**B**) The time from NEBD to anaphase for the indicated conditions as in **A**. Each circle represents the time a single cell spending in mitosis, and the red line indicates the median time. Number of cells analyzed per condition is indicated on top. Pooled data from three independent experiments. Mann–Whitney U-test was applied. ns, not significant. **P < 0.01. (**C**) The histone marker was used to analyze alignment defects upon mitotic exit or delay in formation of metaphase plate. Each column represents the percentage of cells with alignment defects from one experiment. Shown are results from three independent experiments. The error bars represent SEM. (**D**) Representative images of nocodazole-arrested HeLa cells complemented with the indicated YFP-tagged BubR1 constructs (wt or MadBub) after depletion of endogenous BubR1 and stained with DAPI and antibodies for YFP, H2ApT120, and CENP-C. (**E**) Quantification of kinetochore/centromere signal of H2ApT120 normalized to that of CENP-C. At least 150 kinetochores from 10 cells were quantified and plotted for each condition. Bar indicates mean, and SEM is shown by a line. The experiment was repeated twice, and the result of one experiment is shown. (**F** and **G**) Quantification of kinetochore/centromere signal of hSgo2 normalized to that of CENP-C. The experimental design and statistical analysis were similar to **D** and **E**. Scale bar, 5 μm.

Our analysis of Bub1 functional domains revealed an important function of the kinase domain in chromosome alignment. Given our results on engineering PP2A-B56 binding sites into Bub1, we next asked whether there was a requirement for having Bub1 kinase activity restricted to Bub1. To test this, we replaced the BubR1 kinase domain with the Bub1 kinase domain in its active or inactive form (the engineered protein encodes BubR1 1–731 aa fused to Bub1 734–1085 aa and is referred to as MadBub wt or MadBub kinase dead (KD), respectively, as in Suijkerbuijk et al. (2012a)). We investigated the ability of these fusion proteins to support mitotic functions by depleting BubR1 by RNAi and complementing cells with RNAi-resistant YFP-tagged versions. Analysis of mitotic timing in unperturbed cells revealed a slight increase in MadBub wt-complemented cells, and this increase was dependent on a functional Bub1 kinase domain (Figure 4A and B). This mitotic delay correlated with an increase in alignment defects arguing that MadBub wt does not efficiently support proper chromosome alignment (Figure 4C). The effect on mitotic timing might be underestimated, as we observed a slight impairment in checkpoint activity in MadBub wt cells possibly due to reduced formation of MCC (Supplementary Figure S4A and B).

To investigate potential molecular defects causing chromosome alignment defects in MadBub wt-complemented cells, we first stained mitotic cells for H2ApT120, a known Bub1 phosphorylation site. Strikingly, the staining was delocalized throughout chromosomes in MadBub wt cells and no longer focused to centromeres. However, the total levels of centromeric H2ApT120 were not changed (Figure 4D and E). H2ApT120-delocalized phosphorylation was further increased when Bub1 was depleted in MadBub wt-expressing cells (Supplementary Figure S4C). We note a small reduction in Bub1 kinetochore levels in MadBub-expressing cells, but this reduction is unlikely to explain the spreading of H2ApT120 (Supplementary Figure S4D). As shugoshin protein localization to centromeres depends on H2ApT120, we stained cells for hSgo2 and quantified centromeric localization. We observed a 50% reduction in centromeric hSgo2, specifically in MadBub wt-expressing cells (Figure 4F and G). We have not been able to identify an hSgo1 antibody that is suitable for immunofluorescence, but anticipate that hSgo1 localization might also be affected. Although shugoshin proteins are required for proper localization of the chromosomal passenger complex (CPC), we did not observe any changes in the centromeric localization of the CPC component INCENP (Supplementary Figure S4E).

Our analysis of MadBub shows that engrafting a functional Bub1 kinase domain onto BubR1 is not compatible with proper chromosome segregation, which could be due to delocalized H2ApT120 impacting on proper centromeric protein localization. Potentially, our engineered MadBub as part of the diffusible MCC complex allows for delocalized phosphorylation events.

## Discussion

Here we provide important insight into the spatial positioning of phosphatase and kinase activity within the Bub1–BubR1 complex. Our data argue that in human cells the optimal function of the Bub1–BubR1 complex in the SAC and chromosome alignment depends on precise positioning of phosphatase and kinase activity within the complex. Although recruiting PP2A-B56 directly to Bub1 at levels like BubR1 can support chromosome alignment, it disrupts the phospho-dependent interaction with Mad1, hereby impairing SAC activity. This likely explains why the putative B56 binding site in Bub1 is not functional. Since Bub1 T461 is dephosphorylated by PP2A-B56 bound to BubR1 (Qian et al., 2017), this argues that the LxxIxE motif in BubR1 is optimally positioned to balance the activity of the phosphatase in the SAC and chromosome alignment.

Interestingly, in *C. elegans*, Bub1 harbors an LxxIxE motif at position 282–287 aa, a Mad1 binding domain at position 404–419 aa, and a Plk1 docking site at position 526–528 aa, which is highly similar to the arrangement of these modules in Bub1ΔBubR1-B56N. The fact that Bub1 in *C. elegans* contains a functional LxxIxE motif could suggest that phosphorylation of T407, the interaction site for Mad1, is less efficiently dephosphorylated by the bound PP2A-B56 (Bel Borja et al., 2020; Lara-Gonzalez et al., 2021a). This would be in line with our data on Bub1ΔBubR1-B56N, which allows for a functional checkpoint as it does not affect phosphorylation of Bub1 T461. Furthermore, additional contacts between Mad1 and Bub1 in *C. elegans* could make the interaction less dependent on T407 phosphorylation and thus allows for a functional LxxIxE motif in Bub1 (Moyle et al., 2014). However, the levels of PP2A-B56 recruited by Bub1ΔBubR1-B56N appear insufficient to effectively support chromosome alignment in human cells.

In conclusion, the precise spatial position of kinase and phosphatase activity within the Bub1–BubR1 complex is critical for balancing the chromosome alignment and SAC functions of this complex. Determining how the ancestral MadBub protein evolved to either maintain or separate its kinase and phosphatase activities in different organisms and how this balances its different functions in chromosome segregation will be interesting to explore.

## Materials and methods

### Cell culture and RNAi

HeLa or HeLa Bub1KO cells were cultivated in Dulbecco's modified Eagle medium (DMEM) (Gibco) supplemented with 10% fetal bovine serum (FBS) and antibiotics. For RNAi and rescue experiments, cells were seeded in a 6-well plate at ∼50% confluence with thymidine (2.5 mM) added in DMEM. Twenty-four hours later, cells were released from thymidine arrest and transfected with siRNA oligo with RNAi-resistant constructs using Lipofectamine 2000 (Invitrogen). For Bub1 depletion, a second RNAi was performed 24 h later in the presence of thymidine. For BubR1 depletion, thymindine was applied to the medium without a second RNAi. The following morning, cells were released from thymidine arrest and processed for further assays. RNAi oligos targeting Bub1 (5′-GAGUGAUCACGAUUUCUAAdTdT-3′) and BubR1 (5′-GAUGGUGAAUUGUGGAAUAdTdT-3′) or luciferase (5′-CGUACGCGGAAUACUUCGAdTdT-3′) were synthesized from Sigma and used for the RNAi.

### Generation of Bub1 KO cell line

Recombinant Cas9 protein was purchased (GeneArt Platinum Cas9 Nuclease, Thermofisher). gRNA targeting Bub1 exon2 was synthesized according to the instructions with the pre-designed forward primer IVT-TAATACGACTCACTATAGTACAAGGGCAATGACC and reverse primer IVT-TTCTAGCTCTAAAACAGAGGGTCATTGCCCTTGT (GeneArt Precision gRNA Synthesis Kit, Thermofisher). Then, 625 ng of gRNA was incubated with 2500 ng of Cas9 nuclease to form the complex, which was transfected into HeLa cells with Lipofectamine CRISPRMAX reagent. The cells were re-cultivated for single-cell-clone isolation at 24 h after transfection. More than 20 clones were expanded and examined for the presence of endogenous Bub1 by western blotting or immunofluorescence.

### Cloning

The constructs used in this study were constructed using standard restriction cloning methods and mutagenesis by polymerase chain reaction (PCR). Briefly, Bub1 wt or BubR1 wt was cloned into pcDNA5/FRT/TO N-YFP vector by *Kpn*I and *Not*I. To engineer the Bub1ΔBubR1-B56N/C constructs, *Bam*HI site was inserted separately into the Bub1ΔBubR1 cDNA at corresponding positions by PCR. The sequence encoding the region encompassing the BubR1-B56 binding motif (649–697 aa) was inserted using *Bam*HI. To generate MadBub constructs, BubR1 cDNA for N-terminal BubR1 (1–731 aa) was first cloned into N-YFP vector by *Kpn*I and *Bam*HI followed by the insertion of the sequence encoding Bub1 kinase domain (734–1085 aa) by *Bam*HI and *Not*I. To generate a Bub1ΔCENP-F construct, a short sequence for an alternative α helix (5′-TCATCCGAAGAGTACGCTCGTAACTGGGCTGCACTAAAC-3′) was cloned into the Bub1 cDNA to replace the sequence for the C-terminus α helix (1066–1082 aa). The details of cloning will be provided upon request. Gene amplification or mutation PCR was performed with KOD DNA polymerase (Toyobo). All the restriction enzymes were from Thermo Scientific.

### Live cell imaging

HeLa cells were seeded in 6-well plates at confluence ∼50% and synchronized with thymidine. RNA interference was performed as described in the above section. Then, 750 ng of RNAi-resistant YFP-Bub1 or YFP-BubR1 construct and 30 ng of CFP-H3 were co-transfected in each well for undisturbed mitosis imaging. No CFP-H3 was used for SAC assay. The day before live cell imaging, cells were re-seeded into an 8-well chamber slide (Ibidi) with thymidine in the medium. On the day of live cell imaging, cells were released from thymidine 5 h before the start of filming. Leibovitz's L-15 medium (Gibco) containing 10% FBS was applied into each chamber before recording by microscopy. For SAC assays, nocodazole (Sigma) was used at 30 ng/ml. Deltavision Elite system (Cytiva) or Nikon A1HD25 imaging system (Nikon) was used for live cell imaging. YFP and CFP signals were collected every 7 or 10 min for a total of 24 h. Softworx or NIS-Elements AR Analysis was used for data analysis.

### Immunofluorescence, antibodies, and quantification

Cells growing on coverslips were treated as described in the above. Cells were released from the second thymidine arrest into DMEM containing RO3306 (5 μM) for 12 h. Afterwards, synchronized cells were released into fresh medium containing nocodazole (200 ng/ml) for 45 min or MG132 (10 μM) for 105 min. Cells were fixed by 4% paraformaldehyde in PHEM buffer containing 60 mM PIPES, 25 mM HEPES, pH 6.9, 10 mM EGTA, and 4 mM MgSO_4_ at room temperature for 20 min. Then, 0.5% Triton X-100 in PHEM was used to permeabilize the fixed cells for 10 min at room temperature. The antibodies used in this study include Bub1 (Abcam, ab54893, 1:200), Bub1 pSpT (homemade, 1:200), CENP-C (MBL, PD030, 1:800), BubR1 pT680 (Abcam, 200061, 1:500), α-tubulin (Sigma, F2168, 1:400), CENP-F (Abcam, ab5, 1:200), H2ApT120 (Active Motif, 39391, 1:400), Ndc80 pS44, Ndc80 pS69 (kind gift from Jennifer DeLuca, 1:200), and Mps1 pT33 (homemade, 1:200). Fluorescent secondary antibodies are Alexa Fluor Dyes (Invitrogen, 1:1000) except GFP booster-FITC was used for YFP detection (Chromotek, gb2AF488-10, 1:500). Z-stacks with 200 nm intervals were taken with the Thunder Imaging System (Leica) using a 100× oil objective followed by deconvolution with Thunder cleaning function. Signal quantification was performed by drawing a circle around each kinetochore. The three continuous peak values were averaged and subtracted of the background values from a neighboring circle.

### Immunoprecipitation and western blotting

HeLa cells were transfected with YFP-tagged Bub1 or BubR1 constructs. Thirty-six hours later, the cells were treated with nocodazole (200 ng/ml) for an additional 12 h. Mitotic cells were collected by shake off and lysed in buffer containing 10 mM Tris, pH 7.5, 150 mM NaCl, 0.5 mM EDTA, and 0.5% NP-40. For purifications to detect PP2A-B56 binding, a low-salt buffer was used as described previously (Kruse et al., 2013). After centrifugation at 16000 *g* for 15 min, the supernatant was incubated with GFP-Trap agarose beads (ChromoTek) and shaken at 1200 rpm for 30 min at 4°C. After three washes, the bound protein was eluted in 2× LDS sample buffer and applied to sodium dodecyl–sulfate polyacrylamide gel electrophoresis for the detection of indicated proteins. Antibodies used in this study include YFP antibody (homemade, 1:2000), Bub1 pSpT (homemade, 1:1000), BubR1 pT680 (Abcam200061, 1:1000), and B56α (BD, 610615, 1:1000).

### Peptide binding assay

Peptides were purchased from Peptide 2.0 Inc. The purity obtained in the synthesis was 95%–98% as determined by high-performance liquid chromatography and subsequent analysis by mass spectrometry. Prior to ITC experiments, both the protein and the peptides were extensively dialyzed against 50 mM sodium phosphate, 150 mM NaCl, and 0.5 mM TCEP, pH 7.5. All ITC experiments were performed on an Auto-iTC200 instrument (Microcal, Malvern Instruments Ltd) at 25°C. Both peptide and protein concentrations were determined using a spectrometer by measuring the absorbance at 280 nm and applying values for the extinction coefficients computed from the corresponding sequences by the ProtParam program (http://web.expasy.org/protparam/). The peptides at ∼450 μM concentration were loaded into the syringe and titrated into the calorimetric cell containing the B56α at ∼40 μM. The reference cell was filled with distilled water. If the so-called c-value, defined as the ratio of analyte concentration in the cell to K_D_, was <1, a low-c assay was performed using ∼25 μM B56α in the sample cell and peptide concentrations at 1.0–2.0 mM in the syringe. The peptides at ∼450 or 120 μM (for submicromolar affinities) concentration were loaded into the syringe and titrated into the calorimetric cell containing the Plk1 polobox domain, respectively, at 35 or 10 μM.

In all assays, the titration sequence consisted of a single 0.4 μl injection followed by 19 injections, 2 μl each, with 150 sec spacing between injections to ensure that the thermal power returns to the baseline before the next injection. The stirring speed was 750 rpm. Control experiments with the peptides injected in the sample cell filled with buffer were carried out under the same experimental conditions. These control experiments showed heats of dilution negligible in all cases. The heats per injection normalized per mole of injectant versus the molar ratio [peptide]/[protein] were fitted to a single-site model. A 1:1 stoichiometry complex was assumed for the fitting of the ITC binding isotherms in the case of the low c-assays. Data were analyzed with the MicroCal PEAQ-ITC (version 1.1.0.1262) analysis software (Malvern Instruments Ltd).

### Protein purification

B56α was expressed and purified as previously described (Kruse et al., 2013). The Plk1 polo box domain (residues 367–603) was expressed in BL21(DE3) cells overnight at 18°C. Cells were harvested by centrifugation and resuspended in buffer L [50 mM sodium phosphate, pH 7.5, 300 mM NaCl, 10 mM imidazole, 10% glycerol, 0.5 mM TCEP, and 1× complete EDTA-free tablet (Roche)]. Lysis was done using a high-pressure homogenizer (Avestin) and lysate clarified by centrifugation. The clarified lysate was applied to a 5-ml HiTrap Nickle column and proteins eluted with a 10–500 mM imidazole gradient and peak fractions collected. Subsequently, TEV protease was added to remove tag and protein dialysed into buffer L lacking imidazole and loaded onto a 5-ml HiTrap Nickle column and unbound protein collected and pooled. The untagged Plk1 polo box domain was finally loaded on a superdex 200 16/60 column equilibrated with buffer G (50 mM sodium phosphate, 150 mM NaCl, 10% glycerol, and 0.5 mM TCEP) and peak fractions were collected.

## Supplementary Material

mjac062_Supplemental_File

## Notes

**Author contributions:** L.W. cloned Bub1 constructs and performed live cell imaging for SAC and all the immunofluorescence analyses with the help of Y.Z., C.S., and L.Z. T.K. performed immunopurification and analysis of MadBub fusions and live cell imaging of Bub1. B.L.-M. performed ITC measurements. B.L., J.F., and Z.L. provided scientific input and discussed the project with G.Z. J.N. and G.Z. coordinated the work and wrote the manuscript together with T.K.
