# Supplementary material for: Spatial separation of phosphatase and kinase activity within the Bub complex is required for proper mitosis
Source: J Mol Cell Biol. 2022 Nov 28;14(11):mjac062. doi: 10.1093/jmcb/mjac062 (PMC10155811; doi:10.1093/jmcb/mjac062)
Supplement: mjac062_Supplemental_File [file mjac062_supplemental_file.pdf]

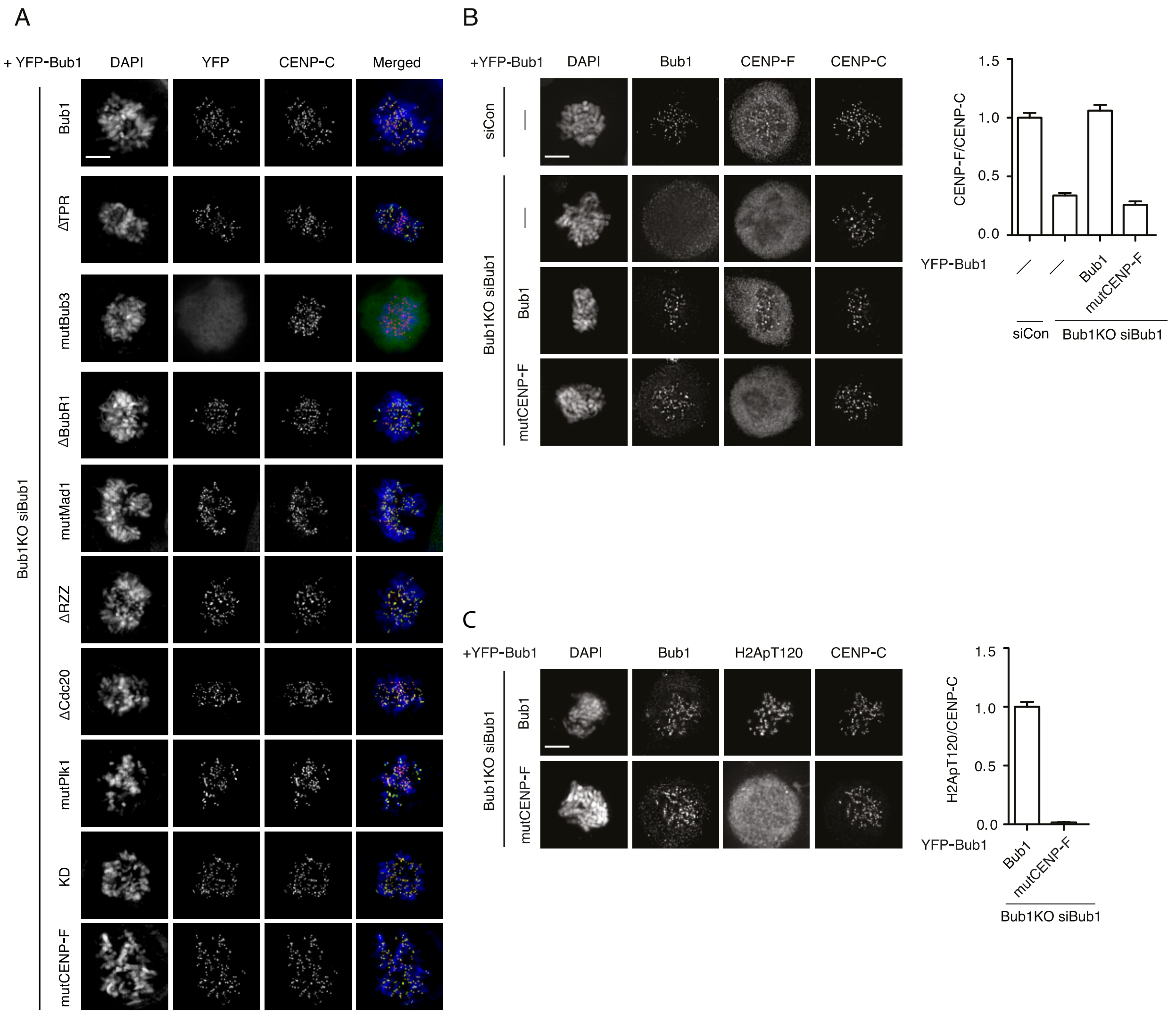

Supplementary Figure S1

Supplementary Figure S1

A) Representative images of nocodazole-arrested Bub1KO cells complemented with the indicated YFP-tagged Bub1 constructs after depletion of endogenous Bub1 and stained with DAPI and antibodies for YFP and CENP-C. In merged images, DAPI is assigned blue with Bub1 green and CENP-C red. The experiment was performed once.

B) Representative images of nocodazole-arrested HeLa (siCon) or Bub1KO cells complemented with the indicated YFP-tagged Bub1 constructs after depletion of endogenous Bub1 and stained with DAPI and antibodies for Bub1, CENP-F and CENP-C. The kinetochore levels of CENP-F normalized to CENP-C is shown on the right. At least 150 kinetochores from 10 cells were quantified and plotted for each condition. Bar indicates mean and standard error of mean is shown by line. The experiment was repeated twice and the result of one experiment is shown.

C) Similar to **B** except cells stained for H2ApT120. Scale bars are 5 μm.

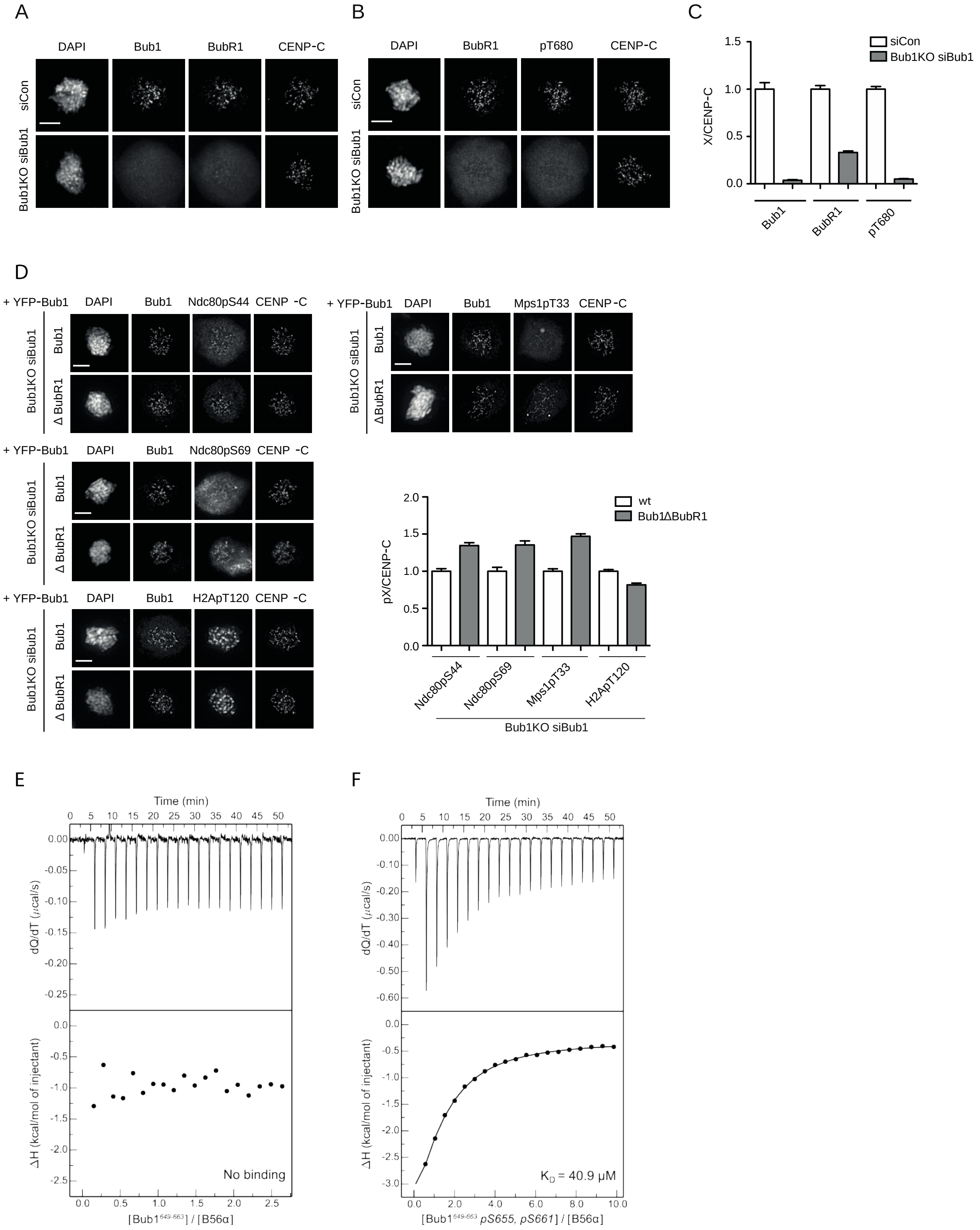

Supplementary Figure S2

Supplementary Figure S2

A) Representative images of nocodazole-arrested HeLa (siCon) or Bub1KO cells after depletion of endogenous Bub1 and stained with DAPI and antibodies for Bub1, BubR1 and CENP-C.

B) Similar as in **A** except BubR1, pT680 and CENP-C were stained in each condition.

C) Quantification of kinetochore levels of Bub1, BubR1, pT680 normalized to CENP-C levels of **A** and **B**.

D) Representative images of nocodazole-arrested Bub1KO cells complemented with YFP-tagged Bub1 constructs after depletion of endogenous Bub1 and stained with DAPI and antibodies for Bub1, CENP-C and the indicated phosphorylation sites. The kinetochore levels of the phosphorylation were normalized to CENP-C. For quantification of **C** and **D,** at least 150 kinetochores from 10 cells were quantified and plotted for each condition. Bar indicates mean and standard error of mean is shown by line. The experiment was repeated twice and the result of one experiment is shown.

E-F) Thermograms for the indicated Bub1 peptides and B56. Scale bars are 5 μm.

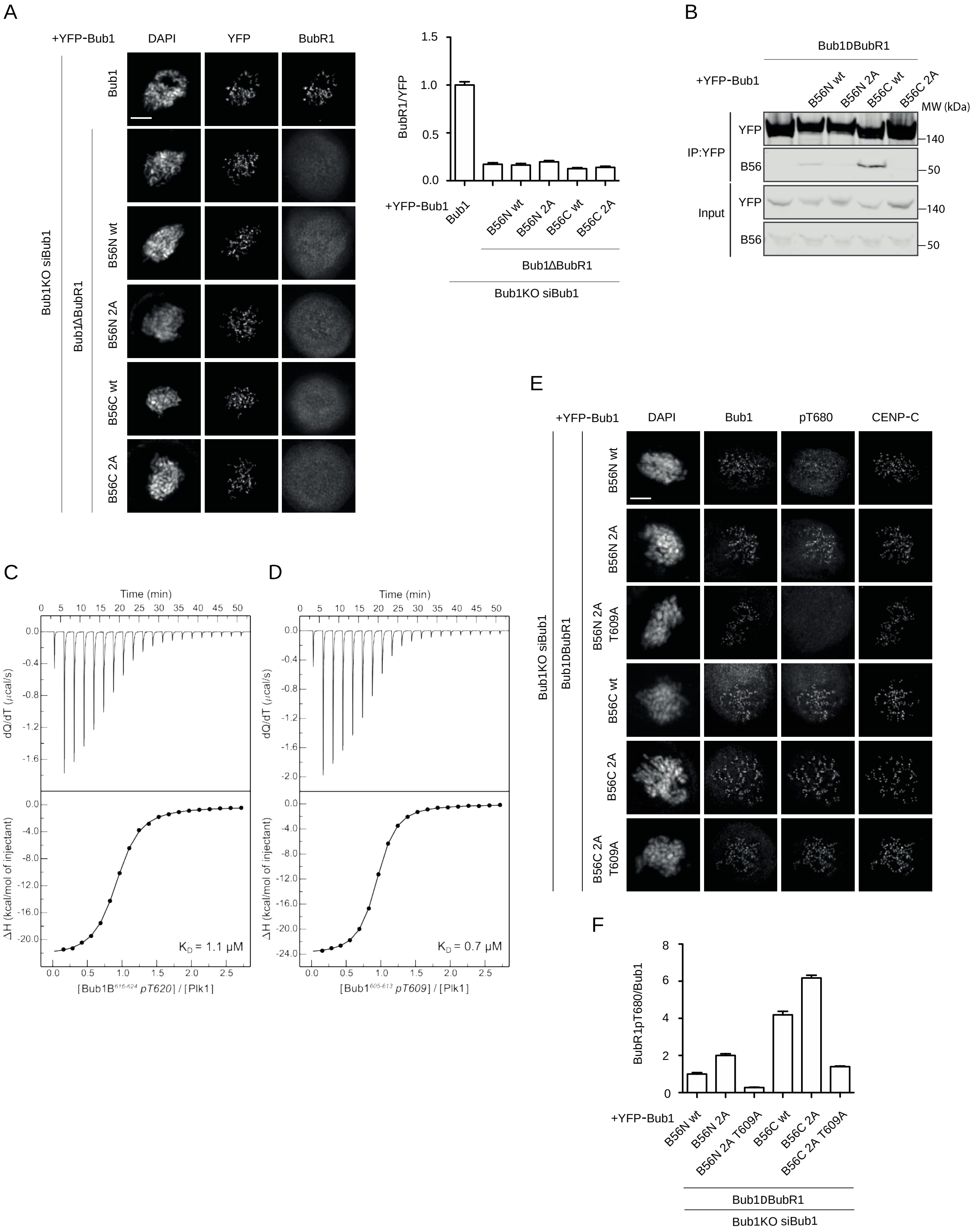

Supplementary Figure S3

Supplementary Figure S3

A) Representative images of nocodazole-arrested Bub1KO cells complemented with YFP-tagged Bub1 constructs after depletion of endogenous Bub1 and stained with DAPI and antibodies for YFP and BubR1. Quantification of kinetochore levels of BubR1 normalized to YFP levels shown on the right.

B) Affinity purifications of the indicated YFP-tagged proteins from nocodazole-arrested HeLa cells. The purifications were probed for YFP and B56α.

C-D) Thermograms for binding of indicated peptides to Plk1 polobox domain.

E) Representative images of nocodazole-arrested Bub1KO cells complemented with the indicated YFP-tagged Bub1 constructs after depletion of endogenous Bub1 and stained with DAPI and antibodies for Bub1, BuBR1 pT680 and CENP-C.

F) The kinetochore levels of BubR1 pT680 normalized to Bub1 from **E**. For quantification of **A** and **F**, at least 150 kinetochores from 10 cells were quantified and plotted for each condition. Bar indicates mean and standard error of mean is shown by line. The experiment was repeated twice and the result of one experiment is shown. Scale bars are 5 μm.

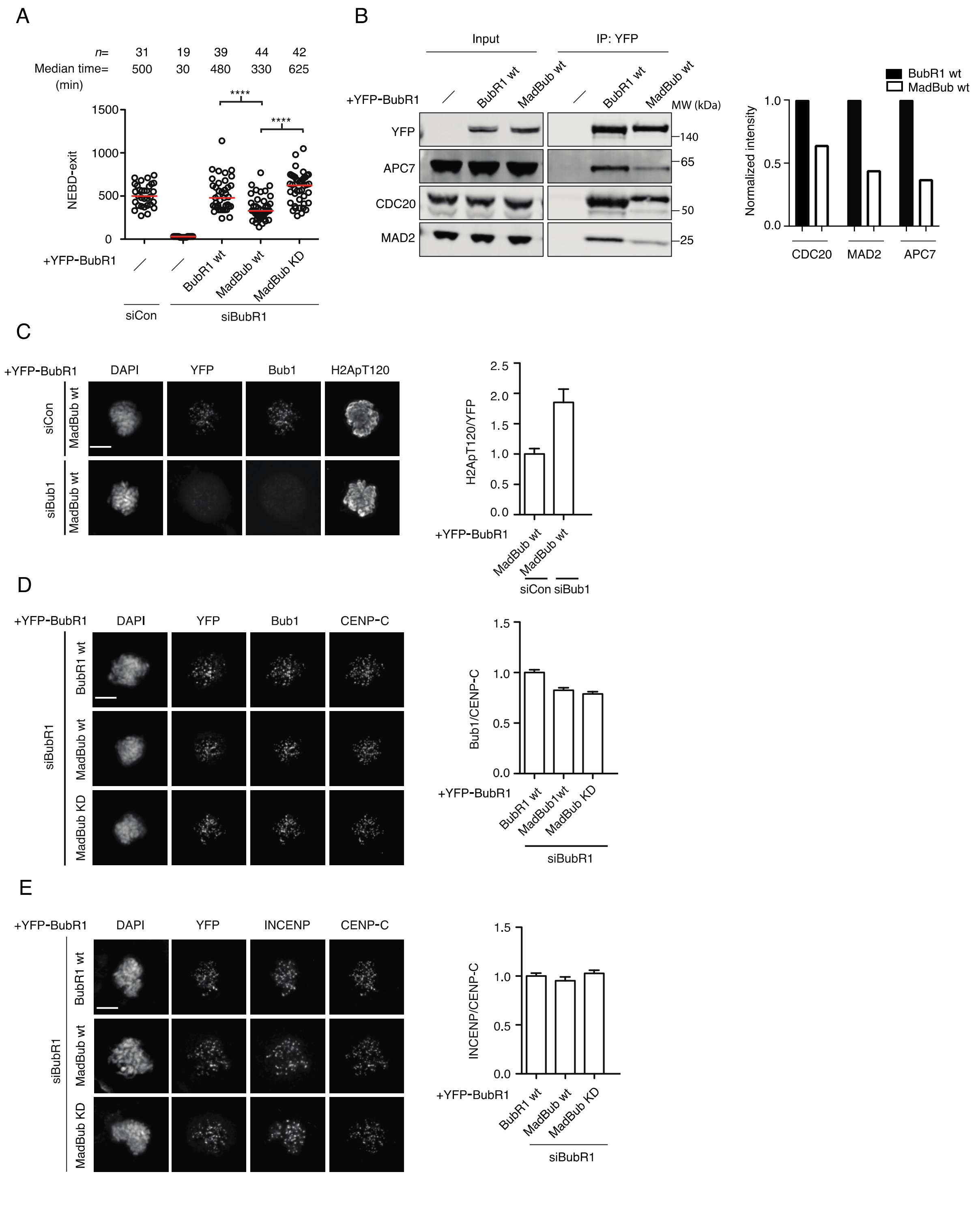

Supplementary Figure S4

Supplementary Figure S4

A) HeLa cells depleted of luciferase as control or endogenous BubR1 and complemented with the indicated YFP-tagged BubR1 constructs (wt or MadBub) were challenged with nocodazole and time from NEBD to mitotic exit was determined by time-lapse microscopy. Each circle represents the time spent in mitosis of a single cell. Red line indicates the median time and number of cells analysed indicated above (n=X). Representative experiment of three independent experiments is shown. Mann-Whitney u-test was applied. ns means not significant. **** means $P<0.0001$.

B) Western blot analysis of YFP immunoprecipitates of the indicated YFP-tagged proteins probed for YFP, APC7, Cdc20 and Mad2. The level of each protein was normalized to that of YFP in the precipitated samples. Quantification of one experiment from two independent repeats is presented on the right.

C) Representative images of nocodazole-arrested HeLa cells depleted of luciferase as control or endogenous Bub1 and co-transfected with YFP-tagged BubR1 (MadBub only) and stained with DAPI and antibodies for YFP, Bub1 and H2ApT120. Quantification of the mean level of H2ApT120 on the whole chromosome surfaces normalized to the mean level of YFP signals in the whole cell shown on the right. 10 cells were analysed and plotted for each condition. Bar indicates mean and standard error of mean is shown by line. Two repeats were conducted and result from one experiment is shown.

D) Representative images of nocodazole-arrested HeLa cells depleted of endogenous BubR1 and complemented with the indicated YFP-tagged BubR1 (wt or MadBub) and stained with DAPI and antibodies for YFP, Bub1 and CENP-C. Quantification of kinetochore levels of Bub1 normalized to CENP-C levels shown on the right.

E) Representative images of nocodazole-arrested HeLa cells depleted of endogenous BubR1 and complemented with the indicated YFP-tagged BubR1 (wt or MadBub) and stained with DAPI and antibodies for YFP, INCENP and CENP-C. The kinetochore/centromere levels of INCENP normalized to CENP-C is shown on the right. For quantification of **D** and **E,** at least 150 kinetochores from 10 cells were quantified and plotted for each condition. Bar indicates mean and standard error of mean is shown by line. The experiment was repeated twice and the result of one experiment is shown. Scale bars are 5 μm.
